# Surface-enhanced stimulated Raman scattering and fluorescence probing of plasmonic nanoparticles in cellular environments: insights into their spatial distribution and aggregation

**DOI:** 10.1039/d5na01029b

**Published:** 2026-02-23

**Authors:** T. Senapati, C. Gerecke, D. Wigger, B. Kleuser, E. Solovyeva, K. Semenov, V. Sharoyko, K. Babich, A. Smirnov, E. Rühl

**Affiliations:** a Freie Universität Berlin, Physikalische Chemie, Institut für Chemie und Biochemie Arnimallee 22 14195 Berlin Germany ruehl@zedat.fu-berlin.de; b Freie Universität Berlin, Pharmacology and Toxicology, Institut für Pharmazie Königin-Luise Str. 2 + 4 14195 Berlin Germany; c Saint Petersburg State University Universitetsky 26 198504 St Petersburg Russia; d Pavlov University L'va Tolstogo 6-8 197022 St Petersburg Russia

## Abstract

Understanding the intracellular distribution of nanoparticles and their cellular uptake is crucial for advancing their theranostic potential, bridging academic studies with medical applications. This investigation examined the intracellular distribution of gold nanobones (GNB) using advanced imaging techniques by comparing results obtained from confocal fluorescence microscopy and stimulated Raman scattering (SRS). GNB show plasmon resonances in the 600–800 nm range and were functionalized with polyelectrolytes and a cyanine 5.5 chromophore to provide both surface-enhanced SRS (SE-SRS) and fluorescence signals, respectively, while exhibiting low cytotoxicity (IC_50_ 4.85 µg mL^−1^). They were modified with folic acid for use in the HeLa cell line. Dual SRS/fluorescence 3D single-cell imaging *in vitro*, supported by scanning electron microscopy, was employed to examine the nanoparticle distribution within single cells, revealing the formation of “hot spots” due to nanoparticle agglomeration. This study underscores the limitations of using GNB for detailed cell imaging and metabolic investigations solely based on either SE-SRS or fluorescence imaging, which is due to the inconsistency of data obtained from either method alone. In contrast, the combined SE-SRS-fluorescence approach revealed detailed information on nanoparticle distribution and clustering within cellular environments, as well as the differentiation of “hot spots”, providing valuable insights into nanoparticle uptake and possible applications in optical diagnostics and molecular biology.

## Abstract 2

Dual 3D imaging with SRS and fluorescence microscopy reveals differences in intracellular plasmonic nanoparticle distribution and “hot spots” *in vitro*.

## Graphical abstract

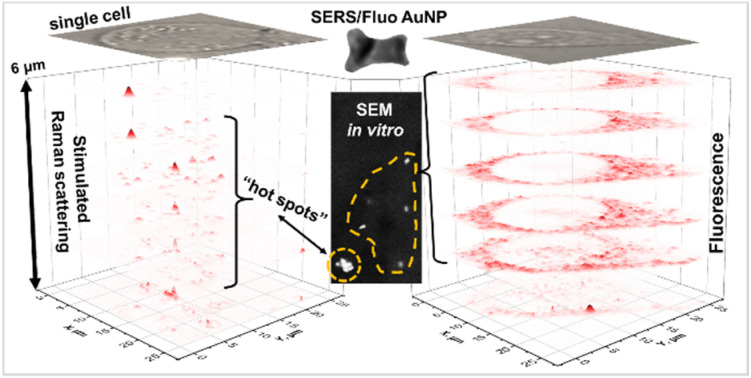

## Introduction

Advancements in fluorescent and Raman tags utilizing plasmon signal amplification have been reported for applications in *in vitro* studies, thin-layer *ex vivo* studies, *in vivo* experiments, and DNA analysis.^1–7^ In the case of spherical nanoparticles, their area of action is often limited to the middle range of the visible spectrum, making them unsuitable for applications such as three-dimensional mapping of objects deep in biological tissues or for express analysis during surgical intervention.^8–10^ To date, numerous studies have been conducted on the synthesis of anisotropic nanoparticles of different shapes that are capable of providing the plasmon resonance band in the 600–800 nm range, which include rod-shaped gold nanoparticles and related structures, such as bipyramids, nanorods, dumbbells, and nanobones.^11–16^ They have already been proven to be active plasmon substrates that provide high optical signal amplification, making them valuable for bioanalytical and medical applications.^17–21^ Light absorbed by gold nanoparticles is converted into heat within picoseconds, which allows for localized heating when using lasers as an excitation source.^22,23^ Thus, plasmonic nanoparticles were shown to have high potential for use in hyperthermal therapy.^24,25^ Since most of the radiation is absorbed by the nanoparticles and dissipated as heat, it is possible to carry out localized overheating of cancer cells, leading to their death.^26^ However, there are distinct challenges in this field, including tracing the location of the particles, low selectivity in the localization of nanoparticles, and limited *in vivo* diagnostics.

Another unique property of gold nanoparticles and plasmonic materials, in general, is their sensitivity to surface-enhanced Raman scattering (SERS), which leads to an enhancement of the Raman signal of molecules located in close proximity to plasmonic surfaces.^27^ Although initially observed on rough surfaces of silver electrodes, it was found that colloidal solutions of gold nanoparticles can also provide SERS.^28–32^ To date, numerous experimental and computational studies have reported how SERS originating from separate non-agglomerated nanoparticles can be exploited in various fields of biological and material sciences, supported by a deep theoretical background.^33–38^

Stimulated Raman scattering spectroscopy (SRS) is another well-known, sensitive, and widely applied approach for label-free biological studies and tissue mapping.^39–42^ It has been used to probe the distribution and binding of biological tags in tissues and biological matrices.^39,43,44^ There are still limited examples of the successful implementation of SRS on nanoparticles, only a few attempts using plasmonic nanoparticles *in vitro* in combination with SRS, and, to the best of our knowledge, no studies using SRS in 3D mode or in a multimodal way for tagged plasmonic nanoparticles.^41,45–49^ Such a method may serve as a valuable approach for gaining insights into the distribution of nanoparticles and their degree of agglomeration in biological media. Complementary information may be obtained by electron microscopy, which is limited to the localization of nanoparticles on or near the surface of cells and requires ultramicrotomy, which is challenging while preserving the structure of single cells and may also require their fixation and dehydration.^50,51^

A recent study showed that SRS imaging of 4T1 cancer cells at Raman shifts of 2852 cm^−1^ (lipids), 2930 cm^−1^ (proteins), and 2968 cm^−1^ (DNA) can be successfully performed.^52^ Another study demonstrated the ability to track viruses in cancer tissues.^53^ Raman spectroscopy was found to be applicable to *in vivo* biodistribution studies on BaSO_4_, which was confirmed by transmission electron microscopy.^54^ Alkyne bond-containing compounds were extensively exploited by spontaneous and stimulated Raman spectroscopy *in vitro* based on probing the vibrational mode of the C≡C triple bond, which is located in the range of 2100–2250 cm^−1^.^46,55^ Terminal alkynes show a Raman signal at approximately 2100 cm^−1^, while internal alkynes show a signal at approximately 2200 cm^−1^, which is separated from other vibrational transitions.

Spontaneous SERS was utilized to study the mechanism and kinetics of an alkyne-tagged acyloxymethyl ketone, a cathepsin B inhibitor.^55^ This approach was based on the appearance of a characteristic signal when the non-tagged drug was co-localized with gold nanoparticles. Since plasmonic nanoparticles can enhance the Raman signal of molecules that are localized nearby, the endogenous chemical surroundings of cells may be studied based on fingerprint shifts of these molecules. However, most of the existing results on 2D fluorescence or SERS mapping on gold nanoparticles lack sufficient spatial resolution, making it difficult to determine the localization and properties of plasmonic nanoparticles.^56–60^ These studies lack a comparative optical and combinational approach, making it difficult to assign differences between SERS and fluorescence data and to determine which methodology provides more relevant information on intracellular distribution. Apparently, *z*-stack SERS mapping revealed aggregation-type behavior,^57^ whereas fluorescence microscopy showed a slightly broader signal distribution for another type of gold nanoparticles.^60^ Nanoparticles were also used for demonstrating surface-enhanced stimulated Raman (SE-SRS) spectroscopy.^61^ An attempt to construct a 3D distribution map was also made, where some clusters were clearly located outside the cells.^58^ The present study aims to fill this gap by providing a methodology for a combined 3D SERS (in SE-SRS mode) and fluorescence approach for distinguishing the differences in optical data obtained *in vitro* at the single-cell level under identical conditions, using the same batch of plasmonic nanoparticles that are probed by SE-SRS and a fluorescence response.

Different strategies have been adopted for the synthesis of surface-modified tagged plasmonic nanoparticles.^62–65^ For example, polyallylamine hydrochloride can be used in combination with anionic polyelectrolytes, such as polystyrene sulfonate (PSS), to form layer-by-layer (LBL) polymeric films.^66^ In general, the LBL technique provides a simple and reproducible way to modify surface parameters, such as zeta potential and biocompatibility, incorporate fluorophores, and attach a delivery vector in comparison with other techniques, such as covalent modifications or growing a silica sealing.^62,67–69^

Among the variety of anisotropic gold nanoparticles, nanobones were chosen for this study since these nanoparticles can provide increased SERS enhancement factors compared to gold nanostars and nanorods of similar absorption parameters.^70^ The chromophores cyanine 5.5 and cyanine 7 were investigated as fluorescent tags and modifiers of silica nanoparticles,^71^ and the resulting particles showed high stability in saline as well as in real blood samples.

Among the derivatives of cyanine 5.5, Cy5.5 amine was found to be the best optical tag for the nanobones in terms of SERS signal intensity, while maintaining its fluorescence emission.^14,70^ Based on these findings, the combination of a gold nanobone core, a cyanine 5.5 amine chromophore, and folic acid as the delivery vector was chosen as the appropriate model system, which is capable of providing surface-enhanced Raman signals and fluorescence in the present investigation.

Cancer cells differ fundamentally from normal cells through their enhanced proliferative capacity, immune evasion, and increased survival mechanisms. These features provide molecular targets for selective drug delivery. One of the key pathways sustaining tumor growth and progression is the uptake of folic acid. The folate receptor, a 40 kDa glycoprotein,^72^ is markedly overexpressed in many cancer cells, where it facilitates folic acid internalization and supports cellular proliferation, migration, and survival.^73–76^ In contrast, normal cells express the receptor at low basal levels,^77^ which is sufficient for essential physiological functions, such as DNA repair, regulation of cell growth and metabolism, and nucleic acid biosynthesis.

Due to its differential expression, the folate receptor has become an attractive biomarker and targeting site for diagnostic and therapeutic applications. For *in vitro* studies of folate receptor-mediated uptake, HeLa cells are commonly employed as a model of folate receptor-positive cancer cells.^78,79^

The combination of SRS and fluorescence microscopy aims to study the 3-dimensional distribution of plasmonic nanoparticles in tumor cells and is supported by transmission electron microscopy. This approach aims to answer the question: How do plasmonic nanoparticles localize after cellular uptake and change their properties of relevance to probing by spectromicroscopy? This study may further facilitate answering the questions of how plasmonic nanoparticles change their properties after cellular uptake, what is the advantage of a combination of Raman and fluorescence approaches, and how it deepens the assignment and understanding of biomedical research.

## Experimental

### Materials

The list of materials used in this work is summarized in the SI (Table S3). All dishes were washed with aqua regia before use and rinsed with MilliQ water.

### Synthesis of gold nanobones

The synthesis of gold nanoparticles in a “bone-like” shape, called gold nanobones (GNB), is based on the procedure for the synthesis of nanorods and differs from the latter in the ratio of the reagents used and the synthesis conditions.^32^ The general synthesis procedure for 400 mL of colloids is as follows: seed solution: CTAB (5 mL) was mixed with 5 mL of 0.1 mM HAuCl_4_, and then 0.6 mL of ice-cold NaBH_4_ was added. The color of the solution changed from pink to cream. Growth solution: separately 200 mL of CTAB solution was mixed with 10 mL of a 10 mM AgNO_3_ solution under vigorous stirring. Subsequently, 200 mL of 1 mM HAuCl_4_ was added. Then, 2.8 mL of ascorbic acid was added to the seed solution (0.552 mL), which discolored the solution. The reaction mixture was then stirred for 48 h in the dark at 25 °C. The final colloid solution appeared dark blue. Washing off the excess CTAB is a mandatory step, as after turning off the heating and stirring, the excess CTAB begins to crystallize. It was shown that if the colloid was heated again and CTAB was dissolved, the plasmon resonance peak irreversibly changed. The resulting colloid was centrifuged in 50 mL tubes for 20 min at 10 000 rpm. The clear CTAB solution was carefully siphoned off with a dispenser to a colloid (0.5 mL), resuspended in deionized water to its original volume, and ultrasonicated for 30 min. The colloid was centrifuged one more time in 50 mL tubes for 20 min at 8500 rpm, the clear CTAB solution was siphoned off with a dispenser from the colloid until 0.5 mL remained, and the colloid was resuspended with deionized water to the desired volume. The resulting colloid was ultrasonicated for 30 min before subsequent use.

### LBL-coating of GNB with encapsulated fluorophore

A scheme for coating with layers of polyelectrolytes was created and experimentally optimized. It is based on several literature sources describing similar methods.^62,80–82^ The purified colloid (100 mL) was added dropwise while stirring to 100 mL of a solution containing 2 g per L PSS and 6 mM NaCl. The mixture was stirred for ∼24 h. The resulting product (200 mL) was purified by centrifugation at 8500 rpm, 50 mL in 50 mL tubes, and resuspended from 0.5 mL to 20 mL. The tubes were then kept in an ultrasonic bath for 30 min, and subsequently the colloid was combined and brought to the original volume of 100 mL. Cyanine 5.5 (Cy5.5) chromophores were added dropwise to 10^−4^ M solutions and stirred vigorously for 2 h. One volume of the resulting colloid was added dropwise while stirring to one volume of a solution containing 2 g per L PDDA and was stirred until the next day. The resulting colloid was purified by centrifugation at 7500 rpm, 50 mL in 50 mL tubes, and resuspended from 0.5 mL to 20 mL. The tubes were kept in an ultrasonic bath for 30 min, after which the colloid was combined and brought to its original volume, yielding the product GNB.

### Functionalization of GNB with folic acid

Folic acid (FA) was selected as a low-molecular-weight model vector to evaluate the antitumor applicability of the approach. For the GNB where the modification with folic acid was carried out, 10^−4^ M folic acid was dissolved in bicarbonate buffer and was added dropwise while stirring to a single solution of nanoparticles in a 1 : 1 ratio, thus reaching a final concentration of 5 × 10^−5^ M. The system was incubated for 2 h while stirring, after which excess folic acid was removed by centrifugation. The obtained product was gold nanobones functionalized with folic acid (GNB + FA).

### Characterization of nanobones

The extinction spectra of the colloidal solutions of nanoparticles were recorded in the wavelength range of 190–1100 nm using UV-1800 and UV-2550 (Shimadzu) scanning spectrophotometers in quartz cells with an optical path length of 10 mm. Dynamic light scattering (DLS) was performed using a Zetasizer Nano (Malvern) with a refractive index of *n* = 0.27 + *i* × 5.95, the built-in preset for gold nanoparticles. The viscosity of the solvent was set to that of water (0.887 mPa s) with a refractive index of 1.33. Histograms of the hydrodynamic size and zeta potential (*ζ*) distribution are shown in Fig. S1 for the bare gold nanobones, PSS-coated nanobones and the final products denoted as “GNB” and “GNB + FA”, and the corresponding mean values are presented in Table S4. Transmission electron microscopy (TEM) images of the gold nanobones were recorded using a Zeiss Libra 200FE microscope at an accelerating voltage of 200 kV. To prepare the samples, 10 µL of gold nanobone colloids were drop-casted on top of carbon films and air-dried. Scanning electron microscopy (SEM) images of the nanoparticles were recorded using a Zeiss ORION scanning helium ion microscope at an accelerating voltage ranging between 10 kV and 40 kV in the scanning mode with a backscattered ion detector and an electron beam surface charge compensation system (ion-beam current: 0.1–100 pA). The edge-sharpness resolution was 0.6 nm. The yields of the synthesized nanoparticles were evaluated using atomic emission spectroscopy with inductively coupled plasma based on the quantity of gold. Samples for quantification were prepared by decomposition of nanoparticles in freshly prepared aqua regia, centrifugation to remove the matrix, and dilution of the supernatant with 0.1 M nitric acid. The calibration curve ranged from 0.001 to 100 µg mL^−1^ and was prepared using standard solutions of hydrogen tetrachloroaurate in 0.1 M nitric acid. Emission spectra were recorded using an ICP emission spectrometer (Shimadzu, ICPE-9000) at *λ* = 242 and 795 nm.

## Methods for computational modeling

To assign the observed vibrational bands of cyanine 5.5 amine, quantum-chemical calculations of their normal modes were performed using the B3LYP hybrid functional in the 3-21G basis set. Quantum-chemical calculations were performed using the Gaussian-16 software package^83^ under license from St. Petersburg State University on the computing cluster of the Computing Resource Center of St. Petersburg State University. The calculations started with the optimization of the molecular conformations, which were set manually closest to the literature data for similar compounds.^84,85^

For a comparison with experiments, all fundamental vibrational wavenumbers were visualized using the GaussView program and were multiplied by a factor of 0.97.^86,87^

### Sample preparation for *in vitro* studies

For stimulated Raman and fluorescence spectroscopy, the cells were incubated overnight in 6-well plates filled with coverslips at a seeding density of 0.3 × 10^6^ in standard growth media (DMEM with 10% FBS, 1% l-glutamine, and 1% streptomycin/penicillin). Subsequently, the medium was siphoned out and the units were washed with PBS and filled with 2 mL FBS-deficient growth medium (1% FBS) containing 2 µg mL^−1^ of nanoparticles in three biological replicates and control samples without nanoparticles. After 2 h incubation, the cells were washed twice with PBS, the coverslips were transferred to new 6-well plates, followed by treatment with 4% formaldehyde in PBS for 15 min, and PBS washing to reach cell fixation. The coverslips were removed from the plates, mounted on glass slides, and sealed around the perimeter with an epoxy resin to avoid buffer evaporation and to allow for long-term storage. Cell samples for SEM were prepared in the same manner as for Raman mapping in 6-well plates. After fixation, the samples were subjected to stepwise dehydration, and carbon was deposited on the surface of the cell monolayers according to a previously described protocol.^65^

### Cell cultures and cell viability studies

HeLa cells were used for all cell culture experiments.^88^ Cells were cultured in standard growth medium consisting of DMEM with 1% l-glutamine, 10% fetal bovine serum (FBS) and 1% penicillin–streptomycin.^89^

The cytotoxicity of the nanoparticles was determined using the 3-[4,5-dimethylthiazol-2-yl]-2,5 diphenyltetrazolium bromide (MTT) assay. Briefly, cells were seeded into 96-well plates at a density of 10 000 cells per well. 24 h after seeding, the medium was replaced with standard growth medium containing the indicated concentrations of nanoparticles. The positive controls were treated with 0.001%, 0.05% and 0.01% sodium dodecyl sulphate (SDS). After an incubation period of 24 h, the cells were washed with phosphate-buffered saline (PBS) and treated with 100 µL MTT solution per well (0.5 mg mL^−1^ in PBS) for 4 h at 37 °C. Subsequently, the supernatant was removed and 50 µL DMSO was added. The plates were shaken at 300 rpm for 10 min at room temperature. The optical density at 540 nm was measured using a microplate reader (Tecan) (Fig. 2C).^90^

### Spontaneous surface-enhanced Raman spectroscopy

Surface-enhanced Raman scattering spectra were recorded on a LabRam HR 800 spectrometer and a SENTERRA express Raman spectrometer (Bruker) in reflection geometry using a modified optical microscope (BX41, Olympus) equipped with 40× standard objective (MPLN, Olympus). The excitation line was 632.8 nm at 30 mW (LabRam) and 785 nm at 100 mW (Senterra), respectively. All studies were carried out in 10 mm standard quartz cuvettes (Merck). The accumulation time was 16 s, and the number of accumulations was 10 in 4 repeats (LabRam) or 40 s in 2 repeats (Senterra). Spectroscopic measurements were carried out in the range of 100–1800 cm^−1^ with a fiber-coupled 80 cm Jobin Yvon spectrometer (Dilor triple XY 800, Horiba) using three 600 lines per mm gratings in the subtractive mode and in the wavenumber range of 400–1800 cm^−1^ (Senterra), respectively. Raman spectra were taken at each point twice and reproduced with an Olympus BX41 microscope using the same acquisition parameters to ensure the post-transportation stability of the nanoparticles.

### Stimulated surface-enhanced Raman spectromicroscopy

Stimulated Raman spectromicroscopy (SRS) studies were performed using an Olympus IX83 inverted microscope equipped with a 50× NIR objective (LCPLN50XIR, Olympus). The samples were excited using a picoEmerald laser system (APE) with a pulse width of 6 ps and a repetition rate of 80 MHz.^91^ This laser emits two pulses, one of which is maintained at a fixed wavelength (Stokes pulse, 1064.2 nm). The wavelength of the preceding pump pulse was adjusted according to the desired Raman transition using the signal wave of an optical parametric oscillator (OPO). For these studies, we chose several of the most pronounced Raman modes according to the results from spontaneous surface-enhanced Raman spectrometry studies of nanoparticles in colloidal solutions, which do not overlap with signals from cells (see Fig. 3). To check the reliability of the signal, we also performed mapping at 2930 cm^−1^, which corresponds to the Raman scattering from proteins. The SRS process was measured using the stimulated Raman loss (SRL) detection technique. The Stokes beam was modulated using an electro-optical modulator (EOM) at 20 MHz. Modulations of the transmitted pump beam were detected using a fast photodiode (DET36A, Thorlabs) and a lock-in amplifier (HF2TA + HF2LI Zurich Instruments). To avoid radiation damage to the samples, the laser power was limited to 5 mW and 12.5 mW for the pump and Stokes beams, respectively. Stimulated Raman maps were obtained from scanning the microscope stage (Märzhäuser) at a dwell time at one spot of 160 ms. Possible unwanted background signals, *e.g.*, from cross-phase modulation, were reduced by subtracting background maps recorded at 2500 cm^−1^, corresponding to a pump beam wavelength of 840.6 nm, where no Raman signal is emitted from the cells.

### Software and graphical representation

Graphs and 2D and 3D maps of the optical signal distributions were processed using OriginPro version 2016 (OriginLab), Microsoft Excel, PowerPoint, Notepad, Paint.net (https://Paint.net), Windows 10, SpectraGryph 12, and Fiji (an open-source platform for biological-image analysis).^92^

## Results and discussion

### Properties of gold nanobones

**Fig. 1:**
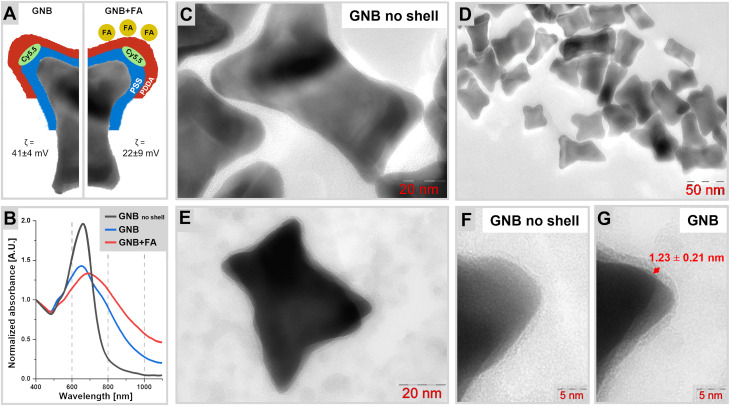
(A) Schematic of the structure of the two types of nanoparticles under study. GNB: gold nanobones, GNB + FA: gold nanobone functionalized with folic acid (FA); cyanine 5.5 (Cy5.5); polystyrene sulfonate (PSS); and poly(diallyldimethylammonium) (PDDA); *ζ* (zeta potential) is provided for GNB and GNB + FA; (B) UV-Vis spectra of GNB after initial synthesis and purification (GNB no shell), GNB, and GNB + FA from (A) normalized at 400 nm; (C–G) TEM images of the synthesized nanobones at different magnifications; and clearly observable external shell of polyelectrolytes around the particle (G) in comparison with the ‘GNB no shell’ (F).

A schematic of the structure of the gold nanobones (GNB) is shown in Fig. 1A. GNB and gold nanobones functionalized with folic acid (GNB + FA) differ only in the presence of electrostatically immobilized folic acid (FA) on their surface. Dynamic light scattering studies of these particles during LbL coating confirmed changes in the zeta potential (*ζ*) from 22 ± 9 mV for bare gold nanoparticles, through −48 ± 8 mV after the polystyrene sulfonate (PSS) coating step, to 41 ± 4 mV following the subsequent addition of a poly(diallyldimethylammonium) chloride (PDDA) layer (Fig. S1 and Table S4). Immobilization of FA onto the positively charged surface, performed in an alkaline sodium bicarbonate buffer *via* the terminal carboxyl group, resulted in a partial decrease in the positive charge to 21 ± 6 mV. In a study, folic acid was co-crystallized with folic acid receptor 1 and it was shown that the terminal carboxyl group does not participate in the interaction with the receptor and remains sterically unrestricted.^93^ The zeta potential of GNB in deionized water was sufficiently positive to ensure stability. A decrease in zeta potential by 10 mV after incubation with folic acid was observed due to the occupancy of the positively charged polycationic sites with folic acid, which proves the successful functionalization of the GNB surface. The synthesized nano-scaffolds were found to have a ‘bone-like’ morphology (Fig. 1C–G) and a length of 40–60 nm. Fig. 1C–E show transmission electron microscopy images of the non-coated GNB at different magnifications. Fig. 1F and G compare the edge morphology of the non-coated GNB and the final GNB after the application of all layers, respectively, with a visible polyelectrolyte shell thickness of 1.23 ± 0.21 nm. The weakly pronounced shell likely corresponds to a thin CTAB layer formed during synthesis.^94^

According to the original methods for the synthesis of gold nanorods, spherical particles with absorption in the 530 nm regime are also formed, which is undesirable.^95,96^ However, the separation of these different architectures was not attempted as the ratio of spheres to nanorods is known to be low.^62,97–101^ In addition to gold hydrogen tetrachloroaurate, this method employs silver nitrate. Thus, one may refer to these particles as bimetallic because silver also participates in the construction of the crystal lattice of the nanoparticles.^102–104^ As a result, only the target particles were formed with an extinction maximum of 660 nm (Fig. 1B). The narrow peak shown in the black curve corresponds to the gold nanoparticles obtained after purification from the excess CTAB. It is noted that the developed synthesis of gold GNB is advantageous in comparison with alternative procedures for the synthesis of nanobones, which often require additional reagents acting as a second surfactant such as benzyldimethylhexadecylammonium chloride or bromosalicylic acid,^16,105^ and it is simpler than elaborate microfluidic approaches yielding similar results.^15^ The introduction of a multi-layered shell (Fig. 1B, blue curve) and subsequent modification with folic acid (Fig. 1B, red curve) caused a redshift in the plasmon resonance of 30 nm. The preservation of one distinct maximum indicates that the dispersion still contains one main fraction of nanoparticles, with some increase in their dispersity detectable by the broadening of the extinction band. Thus, the colloidal system is stable and large aggregates are absent. This is confirmed by dynamic light scattering (DLS) measurements yielding the hydrodynamic radius, indicating the absence of massive agglomeration, while suggesting the presence of dimers or small oligomers. The initial hydrodynamic radius of the bare gold nanoparticles (60 ± 36 nm) was found to remain fairly unchanged after the PSS coating step (59 ± 37 nm) and increased to 106 ± 57 nm and 176 ± 94 nm after the PDDA coating step and subsequent functionalization with folic acid (FA), respectively (Fig. S1 and Table S4). Furthermore, the redshift in the nanoparticle extinction makes not only 632 nm but also 785 nm and 1064.2 nm suitable for Raman studies. Concerning the optical studies, it is noted that DLS measurements rely on a spherical particle approximation, whereas the nanobones are non-spherical and exhibit a certain degree of planarity. This may account for the presence of an additional peak in the 1–8 nm range observed in the size distribution histograms (see Fig. S1).^106^ Furthermore, the deposition of successive polyelectrolyte layers introduces additional limitations to the DLS data by altering the effective refractive index of the gold nanoparticles.

The yield of the final GNB was ∼62%, which is high considering a 3-stage synthesis with two purification steps after each stage (Table S1) and is higher than that of the other methods for the synthesis of nanorods, providing a yield of 30% after the first synthetic stage.^107^ Modification of this synthesis with folic acid led to a lower yield of 24.5% owing to partial accumulation of the nanoparticles in the basic buffer. These conditions are necessary for immobilizing folic acid in the deprotonated form. The pH was brought to neutral after washing before addition to the cell culture.

**Fig. 2:**
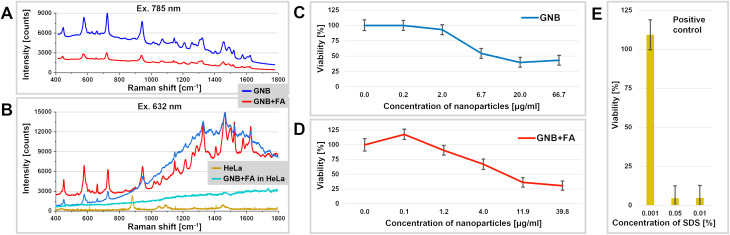
(A) SERS spectra of the gold nanobones (GNB) and gold nanobones functionalized with folic acid (GNB + FA); (B) SERS spectra of GNB, GNB + FA, intact model cell line (HeLa) and after treatment with GNB + FA in HeLa cells; HeLa cell viability test (MTT) in the presence of the indicated concentrations of GNB (C) and GNB + FA (D) at an exposure time of 24 h; (E) positive control cells were treated with 0.001%, 0.05%, and 0.01% SDS. Data are shown as the mean ± SD of 5 independent experiments.

The prepared nanoparticles demonstrated a bimodal SERS-fluorescence response of cyanine 5.5 (Fig. 2A and B), which is achieved through the optimized distance between the fluorophore and the plasmonic core. The positioning of cyanine 5.5 below the first polymer layer allows for the preservation of a sufficiently intense SERS response while avoiding complete quenching of the fluorescence. The dual optical characteristics of GNB and GNB + FA enabled us to utilize these nanoparticle samples for both fluorescence and stimulated Raman imaging to map the nanoparticle distribution within HeLa cells, as detailed below.

### Spontaneous Raman scattering measurements and assignment of Raman shifts in SERS conditions

Prognostic Raman measurements of the obtained dispersions of GNB and GNB + FA showed an intense SERS response originating from the loaded dye (see Fig. 2A). Due to the fact that folic acid is attached to the outer surface of the core–shell structures, its Raman bands do not appear in the recorded SERS spectra, allowing the cyanine 5.5 signatures to be tracked without interference.

For spontaneous Raman measurements, the wavenumbers 1326 cm^−1^ and 1148 cm^−1^ were most suitable for probing the nanoparticles. The former corresponds to the deformational CH-vibrations of cyanine 5.5 amine, while the latter also includes stretching vibrations of the carbon backbone according to results from quantum-chemical modeling. The list of vibrational assignments is given in Table S2. Note that the wavenumber of 1460 cm^−1^ (NC-H_3_ deformation) interferes with the Raman signal of HeLa cells and cannot be used (Fig. 2B).

At the excitation wavelength of 632 nm, both GNB and GNB + FA exhibit an intense fluorescence background originating from cyanine 5.5 amine, which was only partially quenched by the plasmonic nanoparticle surface. The quenching of fluorescence by the plasmonic nanoparticle surface under resonance conditions is distance dependent. Conditions in which fluorescence is only partially quenched, or where the dye molecules are distributed at variable distances from the nanoparticle surface have been reported before.^65,108^

### Viability of cells incubated with GNB

Prior to the *in vitro* spectromicroscopy studies on GNB, a mitochondrial reductase activity-based viability assay was performed (Fig. 2C–E). The right-hand panel of Fig. 2C validates the reliability of the assay through the use of SDS as a positive control, which induced pronounced cytotoxicity at concentrations ≥0.05%.^90^ The viability results demonstrated that both GNB and GNB + FA exhibited minimal cytotoxicity toward HeLa cells (see Fig. 2C and D, respectively). A statistically insignificant increase in cell viability at the lowest concentration of GNB + FA was observed, which is likely due to the stimulatory effect of folic acid on cell proliferation. This became evident under conditions where GNB showed negligible toxicity. The calculated IC_50_ values for GNB and GNB + FA were 5.0 µg mL^−1^ and 4.8 µg mL^−1^, respectively, indicating their low toxicity and suggesting their potential safety for *in vivo* applications. These findings support the suitability of GNB as theranostic nanoplatforms that are capable of inducing tumor cell damage under laser irradiation while remaining non-toxic to normal tissues under non-irradiated (“dark”) conditions.

### Stimulated Raman scattering and fluorescence microscopy analysis

For the stimulated Raman measurements, the pump laser wavelengths were selected based on the most pronounced vibrational modes according to the SERS signals of the colloids, the laser modulation range of the SRS spectrometer, and the need to avoid the vibrational transitions originating from the cells. The detection of the stimulated Raman signal (SRS) from proteins co-located with the Raman signal from the nanoparticles confirms the presence of the nanoparticles by their capacity to enhance the Raman signal of nearby biomolecules.^109–111^ Furthermore, multiple wavenumbers were considered in the SRS studies to probe the nanoparticles and adsorbed biomolecules at 1148, 1326, 1460, and 2930 cm^−1^, respectively. The 2930 cm^−1^ Raman shift is considered the general standard for CH_3_ stretching vibrations in cellular proteins, as well as for saturated CH_2_ bond vibrations in lipids.^112,113^ The control sample, *i.e.*, HeLa cells not exposed to any nanoparticles, showed no significant Raman signal due to cyanine 5.5 (1148 cm^−1^).

**Fig. 3:**
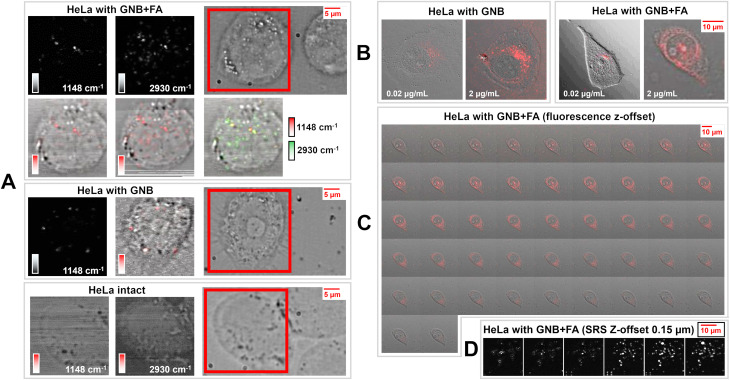
(A) Stimulated surface-enhanced Raman scattering (SE-SRS) 2D mapping. The signal is normalized for each Raman shift separately, non-distinguishable from the background for the HeLa intact sample; incubation time: 2 h and concentration of nanoparticles: 2 µg mL^−1^. (B) Confocal fluorescence microscopy 2D mapping (excitation at 637 nm and emission at 660 nm) of HeLa cells incubated with different concentrations of nanoparticles under the same conditions. (C) Confocal fluorescence microscopy 2D mapping *z*-offset, 3 compilations were used to build a 3D map (see Fig. 4B). (D) SRS 2D mapping *z*-offset (step size: 1 µm) compilation used to build a 3D map (see Fig. 4A).

The Raman signal at 2930 cm^−1^, corresponding to proteins, was considered as the baseline signal for intact HeLa cells. The SRS signals were subtracted from the background measured at 2500 cm^−1^ (Fig. 3A). Alternatively, the GNB and GNB + FA particles in HeLa cells showed an enhancement in the protein signal inside the cells, indicating that the nanoparticles possibly penetrated through the cell membrane *via* endocytosis and were not just immobilized on their surface (Fig. 3A).

The fluorescence images (Fig. 3B) showed that both GNB and GNB + FA were able to stain the cells and provide emission at 660 nm using 637 nm laser radiation for excitation in a concentration-dependent manner, as the same samples with concentrations of 0.02 µg mL^−1^ and 0.2 µg mL^−1^ studied by SRS did not provide any signal that was distinguishable from the background. The peculiar observation obtained from the present study is that the spatial distribution of the fluorescence signal (Fig. 3B) is broader and flatter than the results obtained from SRS (Fig. 3A) for the same samples. The signal also tends to be concentrated around the endoplasmic and nuclear membranes.

Based on these results it may be speculated that GNB + FA were possibly able to bind or permeate the cells more efficiently than GNB without a delivery vector, which is specific for HeLa cells due to the broader signal distribution. However, a separate, more quantitative study is needed, which will include the development of an appropriate set of quantification methods. Only a non-specific endocytosis initiation mechanism is possible for GNB, driven by the electrostatic attraction between the positively charged PDDA-coated nanoparticles and the negatively charged cell membrane. This is followed primarily by clathrin-mediated endocytosis for single nanoparticles and by macropinocytosis, which is more specific for agglomerated nanoparticles.^114–116^ Subsequently, the nanoparticles are expected to be retained in endosomes and lysosomes.

GNB + FA introduce an additional possible mechanism for the initiation of endocytosis *via* receptor-ligand clustering, followed by caveolae- or lipid raft-mediated endocytosis.^117–119^ The resulting vesicles may transport cargo to lysosomes and endosomes. This pathway may allow the nanoparticles to remain in vesicles and lysosomes with a slower acidification rate compared to the non-specific endocytosis of positively charged nanoparticles, thereby shifting the dynamic equilibrium between endocytosis and exocytosis of non-degradable gold nanoparticles toward intracellular accumulation.^120,121^

**Fig. 4:**
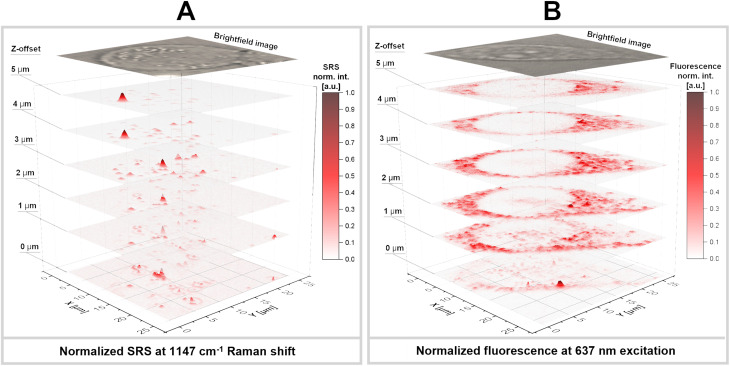
(A) Stimulated surface-enhanced Raman scattering (SE-SRS) 3D mapping. (B) Fluorescence microscopy 3D mapping. The source 2D *z*-offset maps shown in Fig. 3C (several layers are selected with an offset that is identical to 3D SRS). Intensities are normalized within the maximum range of the signal intensity at (A) 1147 cm^−1^ and (B) 637 nm.

The cells stained with GNB + FA were further scanned with a *z*-offset using confocal fluorescence microscopy and stimulated Raman spectromicroscopy (Fig. 3C and D, correspondingly). An attempt to perform the same study with GNB without a FOLR1-mediated delivery vector did not provide the same quality of spatial distribution. This supports the hypothesis of more prevalent accumulation of GNB + FA by FOLR1-positive HeLa cells. Fluorescence mapping revealed that the fluorescence signal intensified while moving through the central plane of the cells. This finding led to the construction of 3D SRS and fluorescence maps based on acquisition parameters optimized for the 2D mode. This was possible for GNB + FA, but not for GNB, possibly due to the lower accumulation of the latter type of nanoparticles in cells. Fig. 4 shows sets of 2D layers with a step size of 1 µm for both SRS and fluorescence scanning (Fig. 4A and B, respectively). Both types of imaging reveal locations of signals on inner layers, which disappear while moving the focal point up or down, where the distribution of intensity differs significantly. This may indicate specific locations where gold nanoparticles agglomerate, creating surface-enhanced stimulated Raman scattering “hot spots” (Fig. 4A). Additionally, single nanoparticles are still present within the cells. However, these single nanoparticles are unable to provide SE-SRS signals, which is consistent with the colloidal suspension of single stable nanoparticles (Fig. 2A and B). The signals from single nanoparticles are evidently not sufficiently strong to compete with the “hot spot”-enhanced signal from nanoparticle agglomerates, while they can be visualized in the fluorescence mode (Fig. 4B). These “hot spots” appear when two plasmon nanoparticles are separated by a distance of a few nanometers, with electromagnetic fields generated by oscillations of electrons, which are induced by the external electromagnetic field of the incident laser beam.^122,123^ The Raman signal from any molecule found near this region is then additionally enhanced up to 10^4^–10^5^ times compared to conventional SERS,^124,125^ while a much weaker SERS signal comes from nanoparticles emitting fluorescence (Fig. 4B), which cannot be observed. On the contrary, significant fluorescence quenching occurs in hot spots because of the enhanced electromagnetic field. This explains why nanobone aggregates are not visible in the images by fluorescence detection.

Another specific observation is that the distribution of the SRS signal overlaps with the localization of cell nuclei, while it is clearly visible that there is no fluorescence from the nuclear regions (Fig. 4A and B, respectively). To date, there is no evidence supporting the ability of gold nanoparticles of the diameter used in this study to permeate cell nuclei.^126^ However, while one study reported that gold nanoparticles larger than 10 nm were unable to enter the nucleus, another study demonstrated that folic acid-modified nanoparticles with a diameter of 21 nm were capable of nuclear entry.^127^ Thus, functionalization might facilitate nuclear uptake where nanobone aggregates appear to contribute to the SE-SRS signal, while fluorescence is quenched. However, it cannot be excluded that these aggregates of nanoparticles are found in nuclear envelope folds near the nucleus,^128^ as the vertical resolution of Fig. 4 is limited.

The observed Raman scattering signal from agglomerates of nanoparticles, which are formed in a non-guided way, *i.e.*, opposed to techniques such as DNA-origami, is due to a combination of scattering from dyes, located within and outside of hot-spots areas.^129–131^ However, since no SE-SRS signal from non-agglomerated nanoparticles was observed, the scattering signal can be attributed to agglomeration-induced SE-SRS. We also note that the average signal-to-noise ratio (SNR) of all the mapped signals with respect to the background is observed to be enhanced by more than a factor of two. This SNR represents the SE-SRS intensity obtained from agglomerates compared to the expected SE-SRS signal from single nanoparticles, which can be probed by fluorescence microscopy and is indistinguishable from the background in Raman mapping. Thus, the agglomeration-induced enhancement factor adding to the SE-SRS enhancement is at least 10^2^, as follows from the recorded signal strength of the same samples.

### Scanning electron microscopy (SEM) of GNB-treated cells

**Fig. 5:**
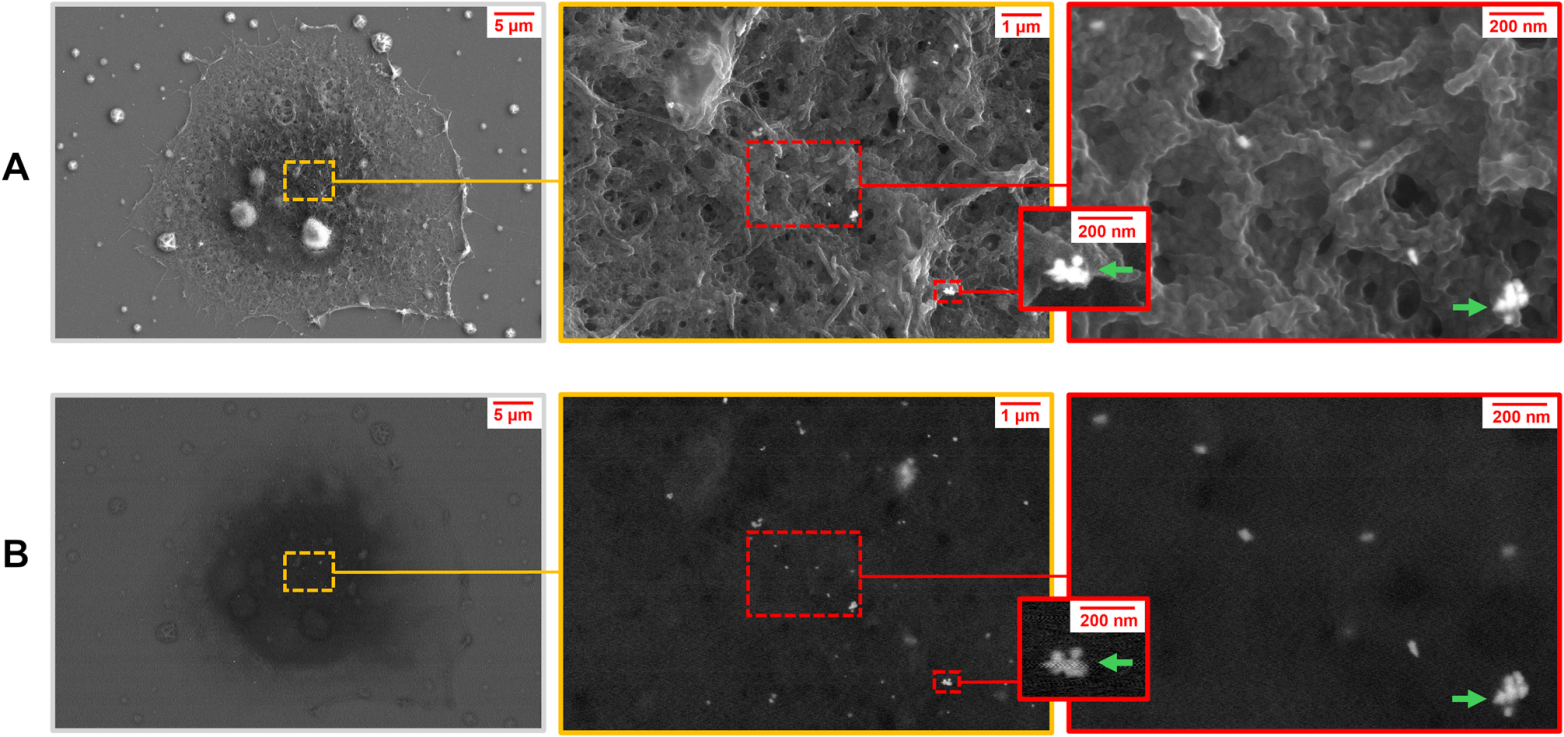
Scanning electron microscopy images of a whole HeLa cell (A) taken in the SE2 mode yielding topographical information. This reveals the distribution of GNB on the surface of the cell (B) taken by backscattered electron imaging (A × B mode). The presence of single nanoparticles, which may provide fluorescence may be distinguished from agglomerates, which are rationalized to be responsible for distinct the “hot spots” in the SE-SRS images (Fig. 4A). Aggregates of GNB are indicated by green arrows.

Scanning electron microscopy was used as another reference method to further confirm the presence of both single nanoparticles and nanoparticles in agglomerates, which may be responsible for the “hot spot” enhancement. HeLa cell samples treated with GNB prepared under the same conditions were fixed with formaldehyde, dehydrated, and stained according to the protocol described in the Experimental section. These samples were analyzed by SEM (Fig. 5). The detection of electrons in the secondary electron mode (SE2 mode) allows for the identification of the topological morphology of the cells with certain areas of high scattering intensity (Fig. 5A). Backscattered electrons taken in the A × B mode reveal the presence of heavy atoms in GNB, and their elongated irregular shape can be recognized (Fig. 1C and 5B). Scanning electron microscopy identified the presence of GNB + FA not only as single nanoparticles but also as several agglomerate clusters of nanoparticles distributed near the surface (highlighted with a green arrow, Fig. 5B). These results can be rationalized by endocytosis leading to the uptake of both aggregates and individual nanoparticles. This may likely correspond to the sources of the “hot spot” signals, which induce a strong surface-enhanced stimulated Raman signal.

## Conclusions

This study demonstrated the synthesis and application of novel tags based on gold nanobones (GNB) with plasmon resonance absorption in the 600–800 nm range for cancer cell targeting. The structure of the optimized tags with the cyanine 5.5 chromophore encapsulated in a polyelectrolyte shell and with folic acid attached to the surface enables their use for fluorescence and stimulated Raman scattering (SRS) imaging. GNB were taken up by HeLa cells expressing folate receptors and exhibited low cytotoxicity (IC_50_ of ∼4.8 µg mL^−1^), allowing their use in medical applications. In addition, receptor-mediated endocytosis may also play a role. These tags may have significant implications for sensitive and targeted tumor identification, detection of ultralow concentrations of substances in tissues, and plasmon-assisted photothermal therapy within the biologically transparent window.

This work demonstrates the synthesis of novel gold biocompatible nanobones possessing minimal interfering contributions to the signal from human tissues. The results indicate how SRS/SERS and SE-SRS can be implemented as a strategy to probe gold nanobones in a biological environment using signals from surrounding proteins. A significant achievement of this study is the implementation of dual SRS/fluorescence 3D imaging to investigate the distribution of plasmonic nanoparticles within single cells. The confirmed formation of agglomerates with “hot spots” provides additional enhancement of the SE-SRS signal by two-orders of magnitude.

This investigation emphasizes the importance of utilizing multiple imaging techniques, as the distribution of fluorescence signals was found to be broader and flatter than the SRS results for the samples under study. The revealed invisibility of single nanoparticles in the SR-SRS images due to insufficient enhancement and their aggregates in fluorescence images due to significant fluorescence quenching is a limitation of the application plasmonic nanoparticles for cell imaging. This finding underscores the complementary nature of these imaging methods and the necessity for a multimodal approach in nanoparticle research that exploits the nonlinear optical features of plasmonic nanoparticles. In conclusion the combined use of SRS and fluorescence imaging, supported by electron microscopy, contributes to an understanding of the specificity of the optical response of plasmonic nanoparticles in biological systems, paving the way for possible applications in medical diagnostics and molecular biology.

## Author contributions

T. S.: investigations, writing the original draft, visualization; C. G., D. W.: investigations, editing; B. K.: providing resources, supervision, editing; E. S.: writing, review, editing, providing resources; K. S.: providing resources, editing; V. S.: conceptualization, supervision, editing; K. B.: data curation, editing; A. S.: conceptualization, synthesis, investigations, funding, visualization, writing the original draft; and E. R.: writing, review, editing, supervision, project administration, providing funding and resources.

## Conflicts of interest

There are no conflicts to declare.

## Supplementary Material

NA-008-D5NA01029B-s001

NA-008-D5NA01029B-s002

NA-008-D5NA01029B-s003

## Data Availability

Additional datasets supporting this work have been included as part of the supplementary information (SI). The datasets generated and analyzed during this work can be made available from the authors on request. Supplementary information: the yield of nanoparticle synthesis, experimental and calculated Raman bands, a list of all chemicals and materials used in this work, and results from dynamic light scattering. See DOI: https://doi.org/10.1039/d5na01029b.
