# Supplementary figures and images for: Surface-enhanced stimulated Raman scattering and fluorescence probing of plasmonic nanoparticles in cellular environments: insights into their spatial distribution and aggregation
Source: Nanoscale Adv. 2026 Feb 23;8(7):2220–32. doi: 10.1039/d5na01029b (PMC12973227; doi:10.1039/d5na01029b)

### GNB uncoated

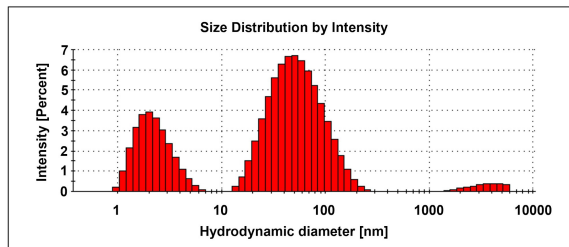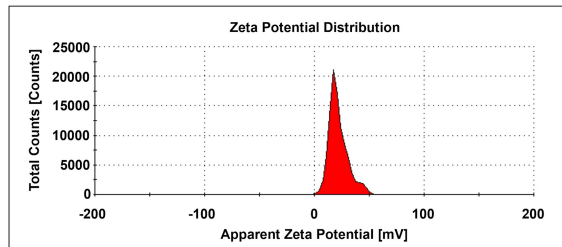

### GNB@PSS

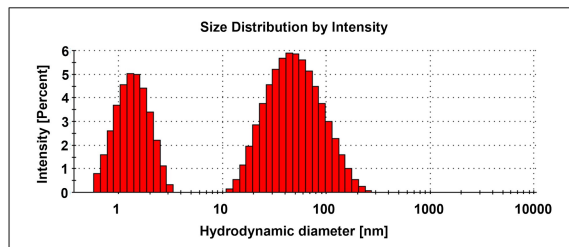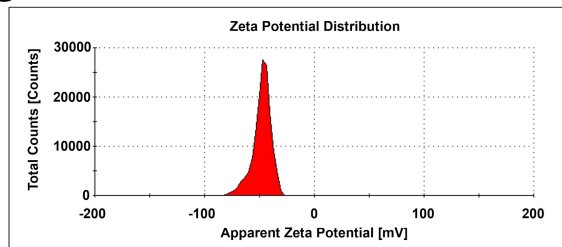

### GNB@PSS@Cy5.5+PDDA<sup>1</sup>

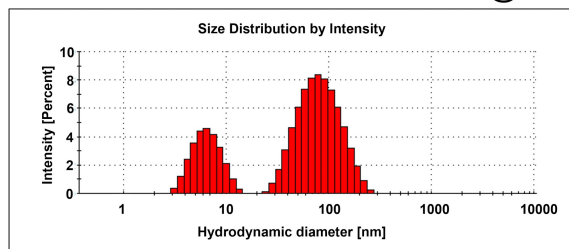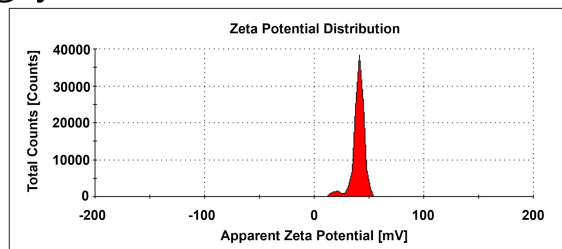

### GNB@PSS@Cy5.5+PDDA@FA<sup>2</sup>

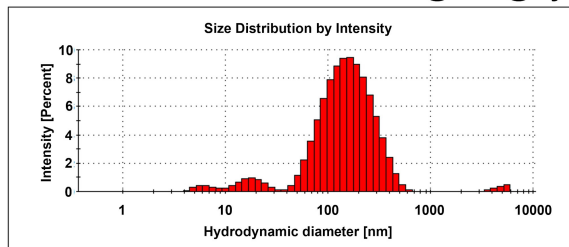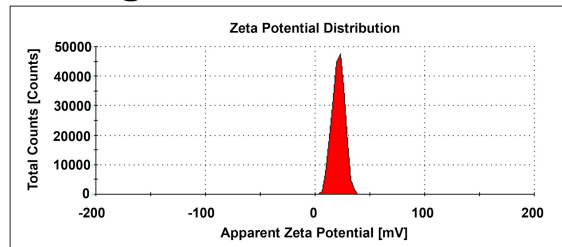

Supplement: NA-008-D5NA01029B-s002 [file NA-008-D5NA01029B-s002.pdf]
