# Supplementary material for: Surface-enhanced stimulated Raman scattering and fluorescence probing of plasmonic nanoparticles in cellular environments: insights into their spatial distribution and aggregation
Source: Nanoscale Adv. 2026 Feb 23;8(7):2220–32. doi: 10.1039/d5na01029b (PMC12973227; doi:10.1039/d5na01029b)
Supplement: NA-008-D5NA01029B-s003 [file NA-008-D5NA01029B-s003.pdf]

Supplementary Information (SI) for Nanoscale Advances.
This journal is © The Royal Society of Chemistry 2026

Supporting Information

Surface-enhanced stimulated Raman scattering and fluorescence probing of plasmonic nanoparticles in cellular environments: Insights into their spatial distribution and aggregation

T. Senapati[a], C. Gerecke[b], D. Wigger[b,e], B. Kleuser[b], E. Solovyeva[c], K. Semenov[c,d], V. Sharoyko[c,d], K. Babich[d], A. Smirnov[c], E. Rühl*[a]

[a] Freie Universität Berlin, Physikalische Chemie, Institut für Chemie und Biochemie, Arnimallee 22, 14195 Berlin, Germany
[b] Freie Universität Berlin, Pharmacology and Toxicology, Institut für Pharmazie, Königin-Luise Str. 2+4, 14195 Berlin, Germany
[c] Saint Petersburg State University, Universitetsky 26, 198504 St Petersburg, Russia
[d] Pavlov University, L'va Tolstogo 6-8, 197022 St Petersburg, Russia
[e] present address: Department of Veterinary Medicines, Federal Office of Consumer Protection and Food Safety, Berlin, Germany

Table S1. The yield of the final nanoparticles synthesis with respect to the theoretical yield assuming that equimolar conversion of hydrogen tetrachloroaurate occurs, as studied by AES-ICP.

	**GNB** $_{calc.}$	**GNB** $_{obs.}$	**GNB+FA** $_{obs.}$
**Conc. [µg/mL]**	106.00	66.72±1.2	39.76±1.2
**Yield [%]**	100.00	62.00±1.2	37.50±1.2

Table S2. Assignment of the experimental Raman modes of GNB according to the quantum chemical calculations of cyanine 5.5 amine. The experimental spectra were denoted with standard notation for Raman spectra, where s – strong, m – medium, w – weak, and vw – very weak bands in relative intensity. Assignment notation: CC, CH, NC, C=C – single and double bonds between corresponding atoms across the molecule. def – deformational, str – stretching modes.

**Experiment**			**Calculation**	
**Raman shift [cm-1]**	**Rel Intensity [A.U.]**		**Raman shift [cm^{-1}]**	**Assignment**
449	180	s	479	CC def
578	313	s	570	CC def
661	76	vw	681	CC def
727	356	vs	719	CC def
945	344	vs	956	CC str, CH def chain
1017	52	vw	1015	CC def ring
1074	117	m	1114	CH def
1131	77	vw	1138	CC str, CH def ring
1148	175	m	1156	CC str, CH def
1164	86	w	1166	CC str, CH def ring
1209	94	w	1178	CC str, CH def
1259	110	w	1252	CH def
1281	94	m	1285	CH def
1307	183	m	1309	CH def
1326	211	m	1328	CH def
1407	50	vw	1396	CC str, CH def ring
1460	239	m	1460	NC-H_3 def
1491	162	m	1498	CH def
1523	171	m	1544	CC str, CH def ring
1578	96	w	1544	CC str, CH def ring
1612	79	w	1619	central C=C str
1626	113	w	1634	central C=C str

Table S3. List of all chemicals/materials used for this work.

Material	Abbr.	Source	CAS No.
Cetyltrimethyl-ammonium bromide, 95%	CTAB	Sigma-Aldrich	57-09-0
Hydrogen tetrachloroaurate, 98%	$HAuCl_4$	Alfa Aesar	16961-25-4
Cyanine5.5 amine	Cy5.5	Lumiprobe	2097714-45-7
Sodium borohydride, 98%	Na_3BH_4	Alfa Aesar	16940-66-2
Ascorbic acid	A.A.	Sigma- Aldrich	50-81-7
Silver nitrate, 99%	$AgNO_3$	Sigma-Aldrich	7761-88-8
Polystyrene sulfonate, 95%	PSS	Sigma-Aldrich	28210-41-5
Poly(diallyldimethylammonium) chloride	PDDA	Sigma-Aldrich	26062-79-3
Dulbecco's modified eagle medium	DMEM	ATCC	30-2002
Fetal bovine serum	FBS	ATCC	30-2020
Trypsin-EDTA 0.25%		Capricorn	TRY-3B
Dulbecco's phosphate-buffered saline	DPBS	Corning	21-031-CV
antibiotic/antimycotic solution (100x)	A/A	Capricorn	AAS-B
Folic acid, 97%		Sigma-Aldrich	59-30-3
Thiazolyl Blue Tetrazolium	MTT	Sigma-Aldrich	298-93-1

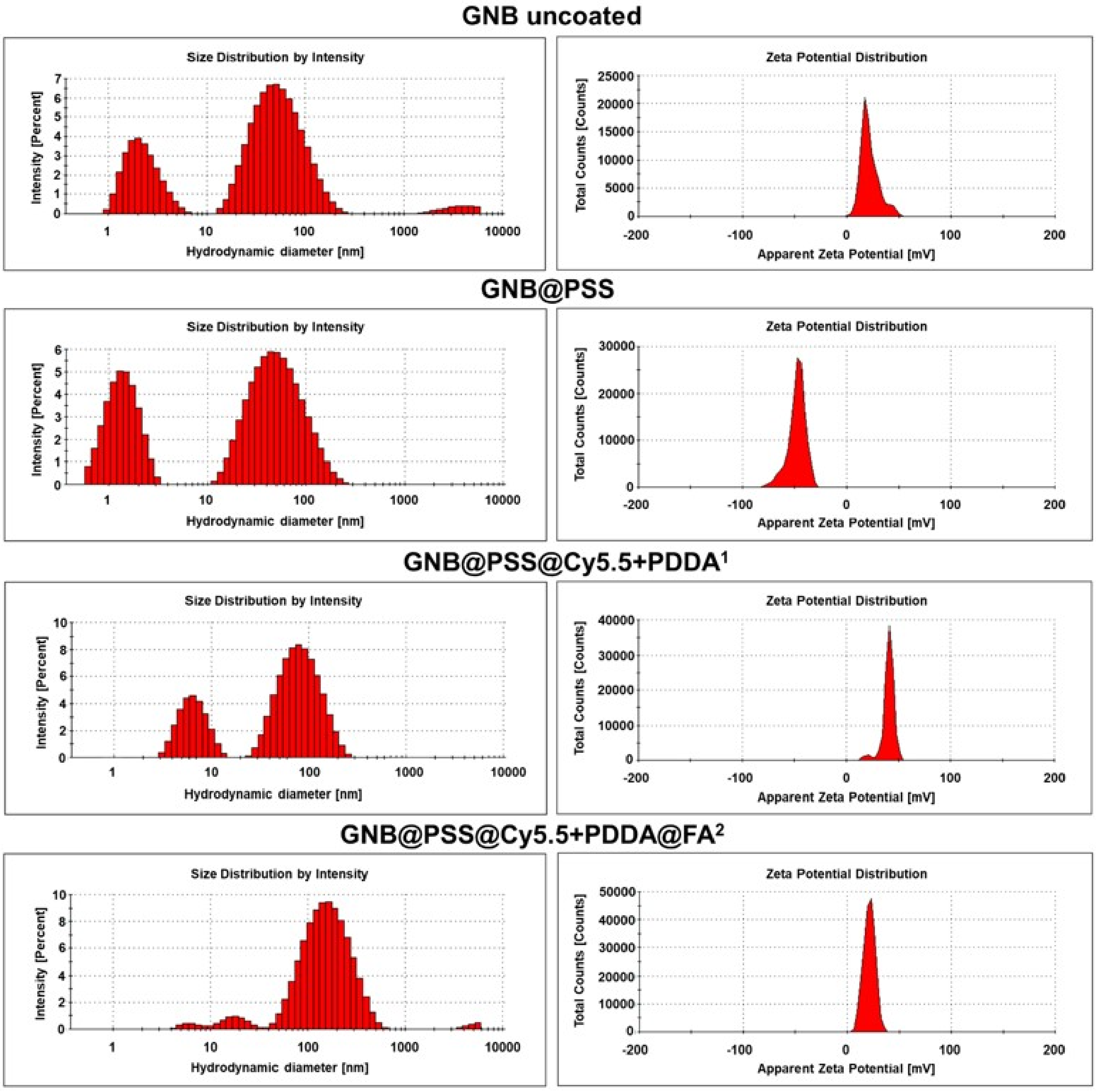

Fig. S1. The hydrodynamic radius and zeta-potential distributions obtained from dynamic light scattering (DLS) experiments. [1]referred as "GNB" in the main text; [2]referred as "GNB+FA" in the main text.

Table S4. Hydrodynamic radius (mean diameter *d*) and mean zeta-potential (ζ) obtained from DLS experiments.

Nanoparticle System	d [nm]	ζ [mV]
GNB uncoated	60 ±36	22±9
GNB@PSS	59±37	-48±8
GNB@PSS@Cy5.5+PDDA[1]	106±57	41±4
GNB@PSS@Cy5.5+PDDA@FA[2]	176±94	21±6

[1]referred as "GNB" in the manuscript; [2]referred as "GNB+FA" in the manuscript
